# *Del1* Knockout Mice Developed More Severe Osteoarthritis Associated with Increased Susceptibility of Chondrocytes to Apoptosis

**DOI:** 10.1371/journal.pone.0160684

**Published:** 2016-08-09

**Authors:** Zhen Wang, Misha C. Tran, Namrata J. Bhatia, Alexander W. Hsing, Carol Chen, Marie F. LaRussa, Ernst Fattakhov, Vania Rashidi, Kyu Yun Jang, Kevin J. Choo, Xingju Nie, Jonathan A. Mathy, Michael T. Longaker, Reinhold H. Dauskardt, Jill A. Helms, George P. Yang

**Affiliations:** 1 Department of Surgery, Stanford University School of Medicine, Stanford, CA, United States of America; 2 Department of Materials Science and Engineering, Stanford University, Stanford, CA, United States of America; 3 Department of Pathology, Chonbuk National University Medical School, Jeonju, Republic of Korea; 4 Palo Alto VA Health Care System, Palo Alto, CA, United States of America; University of Ulm, GERMANY

## Abstract

**Objective:**

We identified significant expression of the matricellular protein, DEL1, in hypertrophic and mature cartilage during development. We hypothesized that this tissue-specific expression indicated a biological role for DEL1 in cartilage biology.

**Methods:**

*Del1* KO and WT mice had cartilage thickness evaluated by histomorphometry. Additional mice underwent medial meniscectomy to induce osteoarthritis, and were assayed at 1 week for apoptosis by TUNEL staining and at 8 weeks for histology and OA scoring. *In vitro* proliferation and apoptosis assays were performed on primary chondrocytes.

**Results:**

Deletion of the *Del1* gene led to decreased amounts of cartilage in the ears and knee joints in mice with otherwise normal skeletal morphology. Destabilization of the knee led to more severe OA compared to controls. *In vitro*, DEL1 blocked apoptosis in chondrocytes.

**Conclusion:**

Osteoarthritis is among the most prevalent diseases worldwide and increasing in incidence as our population ages. Initiation begins with an injury resulting in the release of inflammatory mediators. Excessive production of inflammatory mediators results in apoptosis of chondrocytes. Because of the limited ability of chondrocytes to regenerate, articular cartilage deteriorates leading to the clinical symptoms including severe pain and decreased mobility. No treatments effectively block the progression of OA. We propose that direct modulation of chondrocyte apoptosis is a key variable in the etiology of OA, and therapies aimed at preventing this important step represent a new class of regenerative medicine targets.

## Introduction

Osteoarthritis (OA) represents one of the most prevalent diseases in the United States. It is estimated that 85% of all people reaching the age of 75 will have some clinical evidence of OA.[1] The manifestations of the disease are significant with the symptoms ranging from pain to decreased mobility and disability. Beyond the impact of the disease on the musculoskeletal system, the lack of mobility contributes to exacerbation of heart and metabolic diseases due to decreased ability to engage in physical activity. Current management consists primarily of symptomatic relief ranging from exercise to maintain flexibility and mobility to non-steroidal anti-inflammatory drugs (NSAIDs) for pain control to joint replacement when no options remain. Despite the large number of people affected and the tremendous costs in morbidity, there are surprisingly few alternatives to these therapies.

Hyaline cartilage is unique for its avascular nature and for its limited ability to regenerate. It consists of mature chondrocytes sitting in a highly specialized matrix comprised of glycosaminoglycans that provide the surface required for friction-less motion in the joints. The process leading to clinical OA is believed to be triggered by some form of trauma resulting in inflammation with release of inflammatory mediators and matrix degrading enzymes into the articular space.[2] Among the key inflammatory mediators released is TNFα, a cytokine that promotes apoptosis in chondrocytes.[3] The combination of matrix degradation, chondrocytes apoptosis, and limited regeneration lead to fissures and erosions in the previously smooth articular surface. The primary clinical symptom of this is pain whose severity can lead to disability.

Among humans, there is a clear diversity of susceptibility to the disease. There are 45 year olds with severe enough disease to warrant joint replacement and 75 year olds running marathons. It is apparent that each individual has a different risk for development of the disease. Large-scale population studies looking to identify genetic markers have identified multiple genomic regions indicating that multiple genetic variables contribute to susceptibility.[4–6] Additionally, development of OA is complex and multifactorial with significant influence from environmental factors.

Animal studies have identified a number of genes that might contribute to development of OA and they fall into three broad categories: mutations in extracellular matrix (ECM) and matrix-modifying proteins (COL2A1, ADAMTS5, MMPs)[7–9] that compromise structural integrity, mutations that dysregulate the stress and inflammatory response (HIF-2α, NFκB, IL-1, TNF-α),[3,10,11] and mutations in developmentally regulated proteins (HH, CEBPβ, DKK)[12–14] which adversely affect cartilage development. There have been several mouse mutations of key regulatory genes that exhibit increased apoptosis within the articular chondrocytes in addition to a variety of other effects (SIRT-1, CHOP).[15,16] Mutations in ECM proteins often lead to mice with musculoskeletal abnormalities in the form of chondrodysplasias.[17]

We demonstrate here that DEL1, an ECM-associated, integrin-binding protein, has a potent biological function in chondrocytes where it serves as an anti-apoptotic factor. Furthermore, we show deletion of *Del1* leads to decreased amounts of cartilage as measured by histomorphometry. Knockout mice also have increased susceptibility to OA associated with increased chondrocyte apoptosis.

## Materials and Methods

### Late developmental expression of *Del1 mRNA* and anatomic analysis of *Del1* knockout mice

We used a previously described *Del1-LacZ* knock-in mouse.[18] Identification of areas of expression was performed in heterozygotes at the indicated dates with wild-type (WT) littermates as controls. Specimens were fixed in 4% paraformaldehye and placed in X-gal solution [400 μg/mL X-gal reagent (Invitrogen, Carlsbad, CA), 5 mM potassium ferricyanide, 5 mM potassium ferrocyanide, and 2 mM MgCl2 in 1x phosphate-buffered saline]. Specimens were incubated at 37°C incubator for 1 to 8 hrs until staining was apparent in test specimens but not control specimens, and post-fixed in 4% paraformaldehyde followed by embedding in paraffin, sectioning, and counterstaining with eosin. Characterization of the knockout (KO) phenotype was performed in male, age-matched controls. Knee joints and ears were harvested at 10 weeks of age, respectively, for basic histomorphometry. All animal protocols were approved by the Stanford University Institutional Animal Care and Use Committee in accordance with the NIH Guide for the Care and Use of Laboratory Animals.

### Induction of osteoarthritis, TUNEL staining and immunohistochemistry

8-week old male KO and WT mice underwent surgery to remove the medial meniscus of the right knee. Briefly, mice underwent anesthesia with inhaled isoflurane prior to shaving and prep of the surgical site. An incision was made over the knee followed by resection of the medial meniscus and closure of the incision with 6–0 Vicryl. Mice were recovered under a warming lamp and observed until moving and feeding freely. Post-operative pain control was provided by subcutaneous injection of buprophenone q6 hrs for 48 hrs and as needed afterwards. Euthanasia was performed with CO_2_ inhalation followed by cervical dislocation. All animals survived until the endpoint with no early mortality.

Joints were harvested at 8 weeks after surgery and processed for histology with Safranin O-alcian blue staining. We obtained serial sections of 10 μm across the joint surface and used every third section for analysis resulting in 7–12 sections graded per joint. Grading was performed by a trained pathologist in a blinded manner using the OARSI method of scoring.[19]

TUNEL staining was performed using the *in situ* Cell Death Detection Kit (Roche, Indianapolis, IN). For these studies, mice were harvested at 1 week after surgery. Control was sham operation where the joint capsule was opened without resection of the medial meniscus. We chose site-matched areas to count number of apoptotic cells per high power field.

Immunohistochemistry was performed using antibodies directed to endothelial cells (anti-CD31, BD Biosciences, Franklin Lakes, NJ), lymphocytes (anti-CD45R, e-Bioscience, San Diego, CA), macrophages (anti-F4/80, e-Bioscience, San Diego, CA), and neutrophils (anti-Ly-6B.2, AbD Serotec, Raleigh, NC). Sections from mice 8 weeks after medial meniscectomy were used for angiogenesis and from 1 week after medial meniscectomy for inflammatory cells. For angiogenesis, we counted positive tubular structures per high power field. For immune cells, we counted positively stained cells per high power field. Controls for all immunohistochemistry consisted of incubation without primary antibody and with secondary antibody.

### *In vitro* studies of DEL1 function

Normal human articular chondrocytes (NHACs) (Lonza, Walkersville, MD) in low passage numbers (3–4) were cultured in CGM (Lonza, Walkersville, MD) with 5% FBS and seeded at a density of 5x10^3^ cells/100 μl in 96 well plates coated with 8 ng/mm^2^ Del1 or bovine serum albumin (BSA) coated. Proliferation was assessed by performing WST-8 assays at the indicated times (Sigma-Aldrich, St Louis, MO) and absorbance measured at OD450nm. Attachment was performed by first coating the plates with 8 ng/mm^2^ of BSA or DEL1. NHACs first suspended in CGM with 1% FBS with either 500 μM RGD or RGE peptide, 1:200 dilution of anti-integrin α_v_β_3_ (ab 190147, LM609, Abcam, Cambridge, MA) or IgG1 isotype control, or 1:200 dilution of anti-integrin α_1_ (sc-271034, Santa Cruz Biotechnology, Dallas, TX) or IgG2b isotype control, and incubated at 37°C for 15 min prior to plating. After 6 hrs, unattached cells were washed off and the number of cells attached assayed by WST-8.

Apoptosis was induced with the addition of 10 μM doxorubicin (Sigma-Aldrich, St Louis, MO) or 10 ng/ml each of TNFα/actinomycin D (Sigma, St Louis, MO) in the presence of 500 μM RGD or RGE peptides (Bachem, Torrance, CA). Apoptosis was assayed by caspase 3/7 activity (Promega, Madison, WI). Cell viability was determined by trypan blue exclusion. Anoikis was induced using poly-HEMA coated plates to prevent attachment. NHACs were cultured at a density of 1x10^3^ cells/100 μl in CGM (Lonza, Walkersville, MD) with 0.5% methyl cellulose (Sigma-Aldrich, St Louis, MO) added to avoid survival effects caused by clumping of cells.[20] 250 ng DEL1 or BSA was mixed with suspended chondrocytes for 10–16 hrs and cell survival assayed with trypan blue exclusion.

To examine factors inducing *del1* expression, NHACs were cultured in the presence of recombinant human TNFα (10 ng/ml), IFNγ (10 ng/ml), IL-1α (10 ng/ml), IL-6 (50 ng/ml), TGF-β1 (10 ng/ml), VEGF (100 ng/ml), FGF2 (100 ng/ml) (all from Peprotech Inc., Rocky Hill, NJ) for 24 hr and RNA collected. We performed qPCR on an ABI PRISM 7900H (Applied Biosystems, Foster City, CA) with Cybergreen PCR reagents (Applied Biosystems, Foster City, CA) using primers specific for *Del1* mRNA (forward primer: 5’- CTTTTATCGCCCTTCCCAAGA; reverse primer: 5’- CTTTTATCGCCCTTCCCAAGA).

To obtain primary mouse chondrocytes, 2-week old mice were sacrificed and the femoral head cartilage isolated. Fragments of cartilage were incubated in collagenase solution to obtain single cells. The resulting cellular suspension was centrifuged to pellet the chondrocytes before plating in DMEM with Glutamax (Thermo Scientific, Waltham, MA) and 10% FBS in an incubator at 37°C and 5% CO_2_.

### Biomechanical testing

10 *Del1* KO mice and 10 WT male mice, aged 10 weeks old, were euthanized and the femur was dissected free leaving the femoral head untouched. Tissues were analyzed while fresh, and kept hydrated and moist during the entire testing process. The femur was attached to a support using epoxy glue that was allowed to set for 2 hrs to ensure solid attachment. The stiffness, elasticity and resistance to penetration were measured by a microprobe system in an area on the femoral head toward the greater trochanter. A Keyence VHX-600 microscope was used with the microprobe system to image the sample as well as ensure the consistency of probe placement.

A high compliance microprobe metrology system was used to study the mechanical properties of the articular surface at the micron length scale. The system consists of a steel probe with a flat end mounted on a load cell with mN accuracy to measure the applied force that was used to push against the surface of the test sample. A test sample holder was mounted on a piezoelectric actuator, which allowed displacement control with sub-50 nm resolution. A micrometer-controlled x-y stage allowed the probe to be positioned with < 5 μm accuracy in the plane of the test die and another stage allowed positioning in the z direction in the direction perpendicular to the test die. The piezoelectric actuator was controlled by a LabView program, which allowed both displacement and displacement rate to be controlled. The entire system was mounted on a rigid steel frame to ensure maximum stiffness. 10 measurements for each femoral head were collected for data analysis. Data was further analyzed with Octave software.

### Statistical analysis

For all comparisons of WT and KO animals, the minimum number of animals required for statistical significance was calculated using a significance level (alpha) of 0.05, and a power of 95%. For OARSI scores, statistical significance was calculated using Mann-Whitney *U* test. For *in vitro* and biomechanical studies, statistical significance was calculated using Student’s *t* test with p<0.05 considered statistically significant.

## Results

### Late developmental expression in cartilage

**Fig 1:**
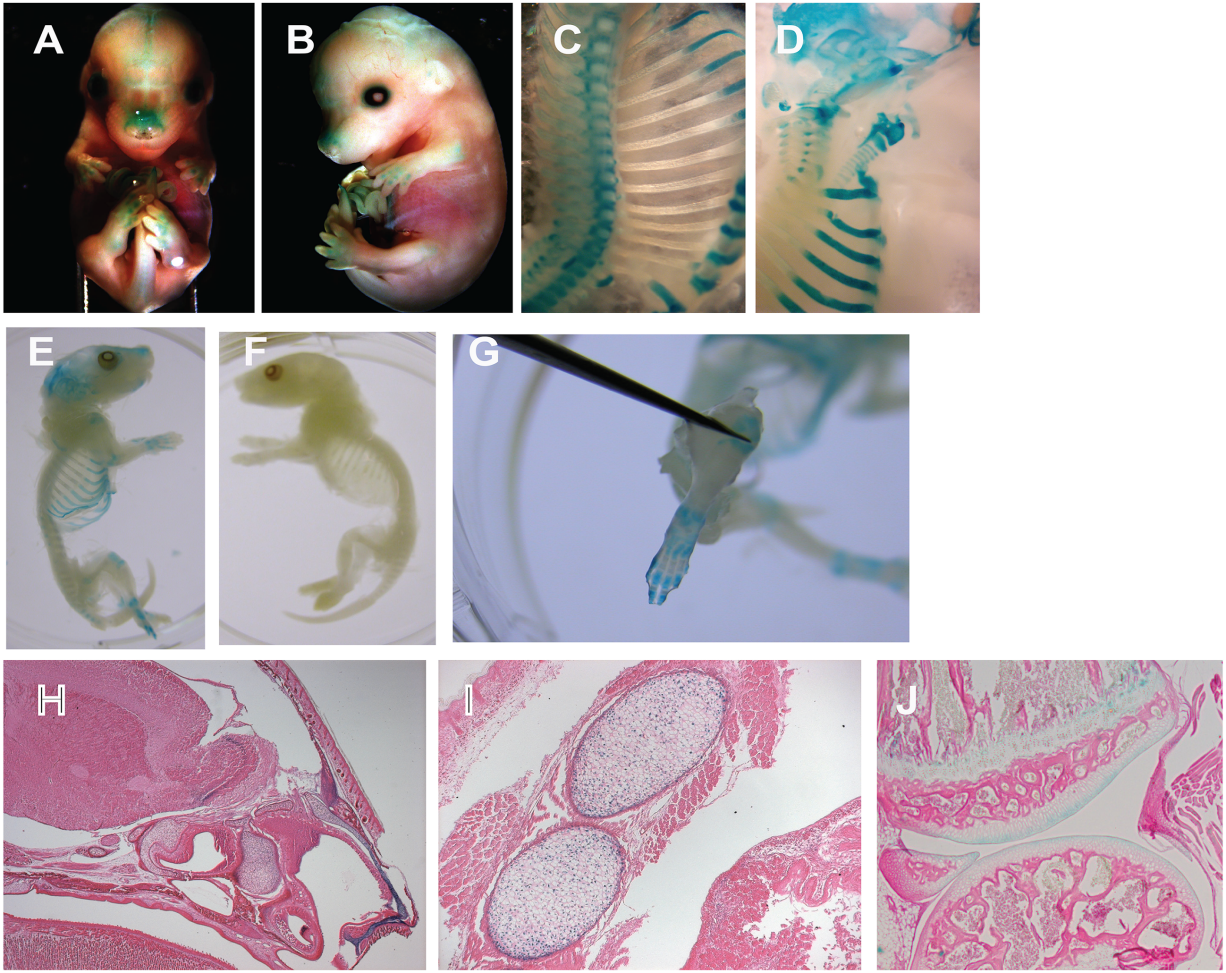
*Del1* developmental expression. Mice at the indicated developmental stage were sacrificed and underwent LACZ staining and whole mount preparation: (A) E14.5, frontal view; (B) E14.5, side view; (C) E17.5, ribs and vertebrae; (D) E17.5, skull base, larynx and trachea, and ribs; (E) D0 transgenic newborn; (F) D0 wildtype newborn; (G) D0 transgenic paw. E17.5 embryos were sectioned, stained for LACZ as indicated by blue staining, and counterstained with hematoxylin. Images are shown of staining in the nasal cartilage (H, 25x magnification), costal cartilage (I, 100x magnification), and knee (J, 25x magnification).

It has previously been shown that *Del1* mRNA was expressed in a variety of tissues during early development including in the hypertrophic cartilage of developing long bone.[18] We looked at expression later during development and in the neonatal period to see if this persisted in mature cartilage. These mice have a *LacZ* gene inserted in the *Del1* gene leading to a knockout of the native gene, and expression of *LacZ* under the control of the native *Del1* promoter. Adding X-gal led to the presence of blue staining wherever LACZ was expressed. *LacZ* expression was found in many diverse areas of mature cartilage, including nose, rib, cranial suture, and trachea comprising both hyaline (joint) and elastic (ear) cartilage (Fig 1). This expression was present in newborn pups as well. Confirmation that the staining was within the cartilage was done with histology (Fig 1H–1J).

### Knockout phenotype

**Fig 2:**
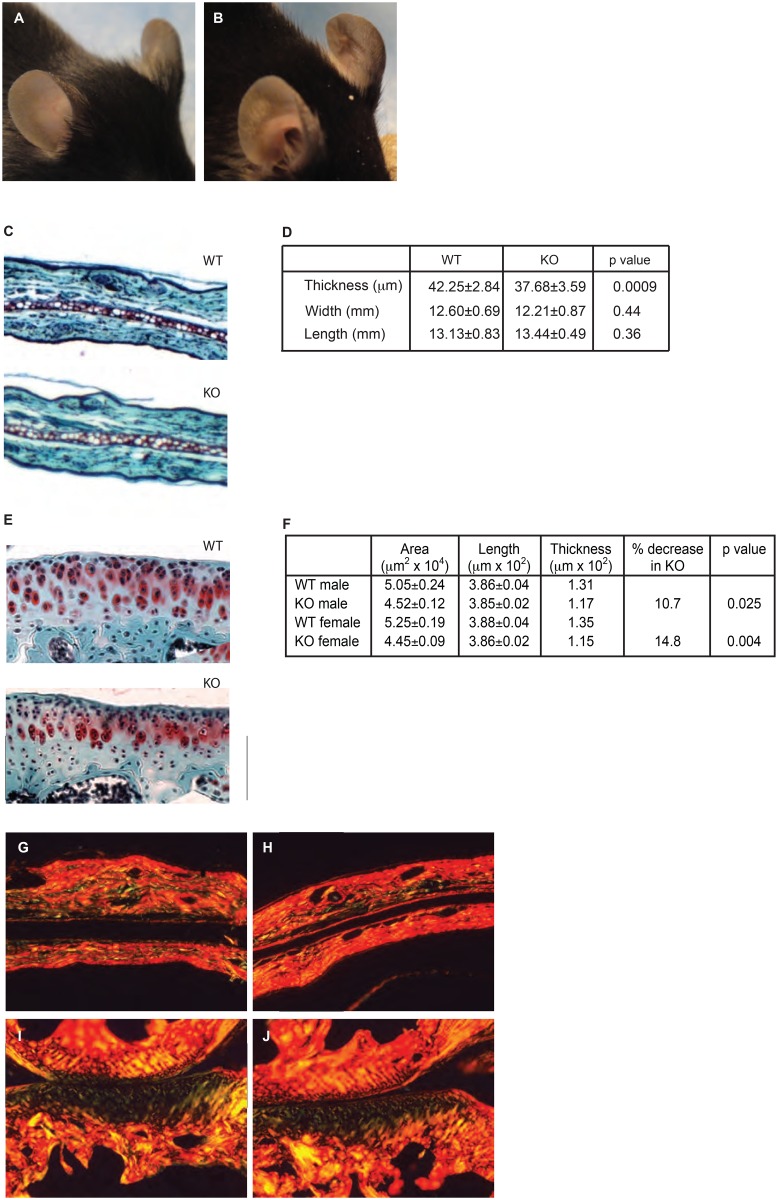
*Del1* KO phenoptype. Appearance of the ear in WT (A) and KO (B) mice. Ears were harvested from 10-week old, male mice and stained (C) for measurement of auricular cartilage thickness (D, n = 12 WT and 18 KO). Width and length are measurements of the pinna. Photomicrographs shown are 40x magnification. Knees were harvested from 10-week old, male mice and stained (E) for measurement of tibial articular cartilage thickness (F, n = 4 for all groups). All values were normalized to tibial length. There was no difference in weight or tibial length between WT and KO mice. A bounding box at 200x magnification as shown was created and the area of cartilage within determined. Due to the variable thickness present within the ear and the undulating boundary between cartilage and bone in the knee, thickness was calculated by measuring the length and dividing into area. p value refers to difference between WT and KO mice. Picrosirius red staining of KO (G) and WT (H) ears and the medial surface of KO (I) and WT (J) knees. Representative sections are shown at 25x magnification.

*Del1* KO mice were born in normal Mendelian ratios, had normal fertility, and activity. Using both plain radiographs and microCT, we found no differences in the bony skeleton either in bone density or morphology. There was no difference in size between KO and WT mice based upon tibial length (1.82±0.01 cm KO vs 1.81±0.02 cm WT, p = 0.24, n = 26 KO, 12 WT). It was noticed that *Del1* KO mice had floppy ears that were most noticeable in the first week of life (Fig 2A and 2B). Cartilage provides the structural framework for the ear and it was hypothesized that there was a difference in the auricular cartilage leading to the phenotype. Compared to age- and sex-matched controls, 10-week old KO mice demonstrated a 10% decrease in the thickness of the auricular cartilage (Fig 2C and 2D). Ear size did not vary as measurements of the length and width of the pinna did not show a difference (Fig 2D) indicating that the ears were not floppier simply because they were larger. Picrosirius red staining for collagens did not demonstrate any substantive difference in the matrix (Fig 2G and 2H). We concluded that the floppier ears in the KO mice were due to decreased amount of total cartilage.

We further investigated whether other sites in the cartilaginous skeleton were affected by *Del1* deletion. Histomorphometric measurements of knee articular cartilage demonstrated that the thickness of the articular cartilage was reduced by 10–15% in the KO compared to the WT mice (Fig 2E and 2F). Given that we found decreased thickness of the articular and auricular cartilage, we reasoned that it could be due to decreased matrix production, decreased chondrocytes, or both. We hypothesized that if chondrocytes produced less matrix, cell density would increase since there is less matrix between them. We examined the cellular density of the cartilage by counting the number of chondrocytes present in a high power field. It was found to be practically identical (2.91±0.08 WT vs 2.89±0.08 KO, p = 0.93). We interpret these results to indicate there is no decreased matrix production, and suggest that the decreased thickness was due to decreased numbers of chondrocytes (S1 Fig). Picrosirius red staining was performed to look for gross differences in collagen content and structure within the cartilage and this demonstrated no difference (Fig 2I and 2J). Although we only surveyed two anatomic sites, the fact that these represented two different types of cartilage, elastic and hyaline, led us to conclude that there was a general decrease in cartilage throughout the skeleton despite no differences detected in the bony skeleton.

### *In vitro* effect on chondrocytes

**Fig 3:**
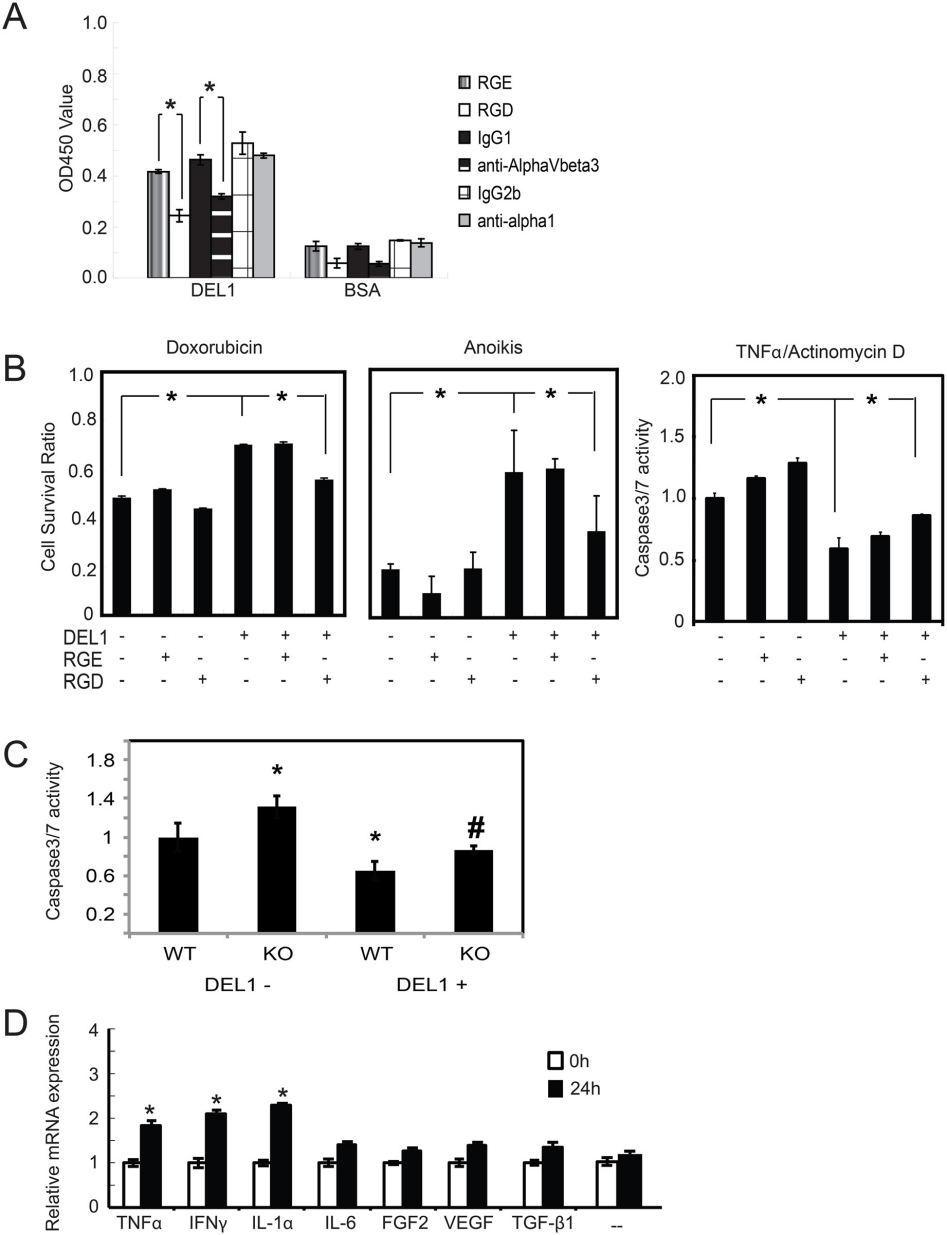
DEL1 effect on apoptosis and induction. (A) NHACs were pre-treated with the peptides or antibodies indicated and placed in plates coated with either BSA or DEL1. Cells attached after 6 hrs were determined by WST-8 assay. *p<0.05 between indicated values. (B) NHACs cultured with DEL1 have increased survival after pro-apoptotic stimuli that were inhibited by RGD, not RGE, peptides. For caspase 3/7 assays, untreated chondrocytes were arbitrarily assigned the value of 1. *p<0.05 between indicated values. (C) Primary chondrocytes from WT and KO mice had apoptosis induced with TNFα/actinomycin D in the presence or absence of purified DEL1 and assayed for caspase 3/7. *p<0.05 relative to WT without DEL1, #p<0.05 relative to KO without DEL1. (D) NHACs were treated with indicated factors (—indicates no treatment). RNA was assayed for *Del1* mRNA expression by qPCR with amount at time 0 without treatment arbitrarily set at 1. Values are average of 3 separate experiments. *p<0.05 relative to untreated cells at 24 hrs.

DEL1 is a matricellular protein as defined by its modular protein structure and its ability to interact with the ECM and cell surface receptors in the form of integrins.[21] Integrin binding can promote a variety of cellular functions including proliferation, attachment and apoptosis. We analyzed the impact of DEL1 on normal human articular chondrocytes (NHACs) to better understand what role it may be serving there. Although we chose to study NHACs, we recognize that mouse chondrocytes or chondrocytes from joints with OA could have different biology. DEL1 promoted chondrocyte attachment via its RGD motif as indicated by effect inhibition of attachment by RGD peptide, but not RGE, and attachment was mediated, at least in part, by integrin α_v_β_3_ (Fig 3A).

We tested for the effect of DEL1 on NHACs after apoptosis was induced through either the extrinsic pathway using TNFα/actinomycin D or via the intrinsic pathway using doxorubicin (Fig 3B) and found it prevented apoptosis of NHACs. The anti-apoptotic effect of Del1 was blocked by RGD peptides indicating that integrin binding was the primary mediator of this effect. DEL1 had no effect on NHAC proliferation (S2 Fig).

Primary mammalian cells commonly need attachment to ECM for survival and the induction of apoptosis due to lack of ECM attachment is termed anoikis. Chondrocytes grown in suspension can avoid anoikis by aggregation due to interactions of cells with the ECM produced by other cells, and this process is integrin-dependent.[22] The addition of methyl cellulose prevents these cellular interactions in suspension and will induce anoikis in chondrocytes. In NHACs grown on polyHEMA-coated plates to force suspension culture and in the presence of methyl cellulose to prevent aggregation, DEL1 was highly protective against anoikis (Fig 3B).

### *Del1* KO mice had increased susceptibility to osteoarthritis

**Fig 4:**
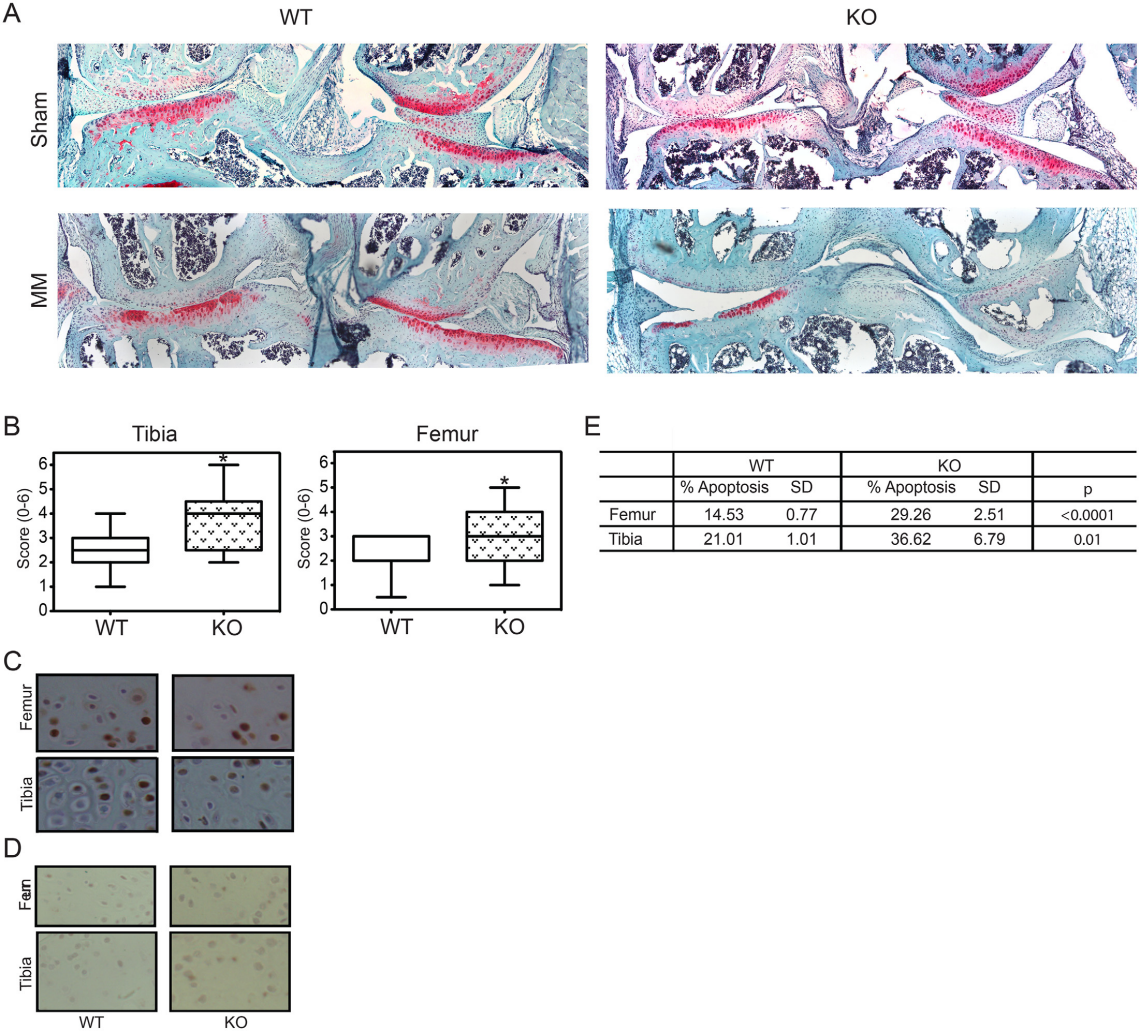
Osteoarthritis susceptibility. (A) 25x magnification view of knee joints from WT and KO mice after sham operation or medial meniscectomy (MM). (B) Box and whiskers plot of histologic scoring of medial tibial and femoral surfaces for OA. *p = 0.0206 for tibia, p = 0.0003 for femur, n = 18 WT and 17 KO. Representative photomicrographs of TUNEL staining of articular surfaces at 1 week after knee destabilization in the injured (C), and sham operated (D) knees. Apoptotic cells seen in the same area of the articular cartilage were counted at 200x magnification as shown and quantified (E). *p<0.001 for femur and p<0.00001 for tibia, n = 5 WT and 6 KO.

As noted above, apoptosis is an important step to developing OA. Because of the significant impact of DEL1 on chondrocyte apoptosis, we predicted that the KO mice would develop more severe OA in response to injury than WT mice. Normal laboratory mice rarely develop OA when allowed to live to relative old age without intervention.[7] We chose to use a model of post-traumatic OA because or relatively rapid and consistent progression of disease to assess whether KO mice had increased severity of disease. We performed a medial meniscectomy to destabilize the knee in 8-week-old male KO and WT mice.[7] Mice were harvested at 8 weeks after surgery and the degree of OA scored by a trained pathologist (KYJ) blinded to the mouse genotype using an established and validated system.[19] Representative photomicrographs of WT and KO mice after medial meniscectomy or sham surgery are shown (Fig 4A). KO mice had significantly worse destruction of the medial articular surface of the tibia and femur as determined by average score for OA severity (Fig 4B). The sham-operated knees had no evidence of OA.

### Exacerbation of osteoarthritis was associated with increased chondrocyte apoptosis

Apoptosis is an early event in the development of OA and precedes histologic evidence of articular surface damage. We hypothesized that we would see evidence of increased apoptosis in *Del1* KO mice early after knee surgery so we harvested a separate group of animals after 1 week to evaluate for the degree of apoptosis within the articular chondrocytes. Using TUNEL staining we found significantly increased numbers of apoptotic cells on the medial tibial and femoral articular surfaces of KO knees consistent with the sites exhibiting the most severe histologic OA (Fig 4B–4E). There was essentially no apoptosis seen in sham-operated knees (Fig 4D). Collectively, these data suggest that DEL1 protein was protective against OA by preventing chondrocyte apoptosis.

We next asked whether chondrocytes from KO mice were more susceptible to apoptosis when compared to WT. We collected primary chondrocytes from the joints of WT and KO mice and induced apoptosis with TNFα/actinomycin D. Chondrocytes were grown in the absence or presence of purified DEL1 protein. WT chondrocytes showed increased resistance to apoptosis with added Del1. KO chondrocytes were more susceptible to apoptosis than WT in the absence of DEL1, and approached WT in the presence of DEL1 (Fig 3C).

### No difference in angiogenesis and inflammation

The development of OA results from the complex interaction of many different cell types. While we could not exclude every other possible cellular mechanism by which DEL1 protects against OA, we did address some of the more relevant possibilities. TGF-β1 was shown to induce high levels of angiogenesis along with increased OA,[23] increased angiogenesis has been reported in the tissues around OA-affected joints, particularly the synovium[24,25] and DEL1 was reported to have angiogenic activity[26] creating the possibility that there were aberrations in angiogenesis around the knee that might have contributed to development of OA. Using immunohistochemistry with anti-CD31 antibodies to assess vascularity, we found no differences between WT and *Del1* KO mice (S3 Fig).

When we examined other protein factors and cytokines that stimulated *Del1* mRNA expression in chondrocytes, we found IL-1α, TNFα and IFNγ, all important inflammatory mediators implicated in OA,[3] significantly up-regulated expression (Fig 3D). Despite its initial identification as an angiogenic factor, *Del1* mRNA was not up regulated by PDGF, VEGF or FGF2 in endothelial cells, or by VEGF or FGF2 in chondrocytes ([27]and Fig 3D).

In addition to angiogenesis, DEL1 facilitates leukocyte recruitment to areas of injury.[28] It was shown that *Del1* KO mice had a greater accumulation of neutrophils in a lung injury model. MFGE8, the only known protein family member of DEL1, aids phagocytosis of apoptotic cells by binding exposed phosphotidyl serines on apoptotic cells through their discoidin-like domain and integrins on macrophages through the RGD motif to facilitate clearance.[29] A similar function has also been ascribed to DEL1.[30] We examined whether there were any differences in the inflammatory response using immunohistochemistry with antibodies directed against lymphocytes (anti-CD45R), macrophages (anti-F4/80) and neutrophils (anti-Ly-6B.2). Counting of positive cells per high power field demonstrated no differences in the presence of the various lineages of inflammatory cells in the injured joint (S3 Fig). There can be changes in immune function that we do not detect with this gross assay, but the papers describing the impact of DEL1 and MFGE8 on immune cell function noted there were differences in immune cell localization due to the effects on diapedesis and phagocytosis.[28,29]

### Cartilage from *Del1* KO mice was biomechanically similar to WT

**Fig 5:**
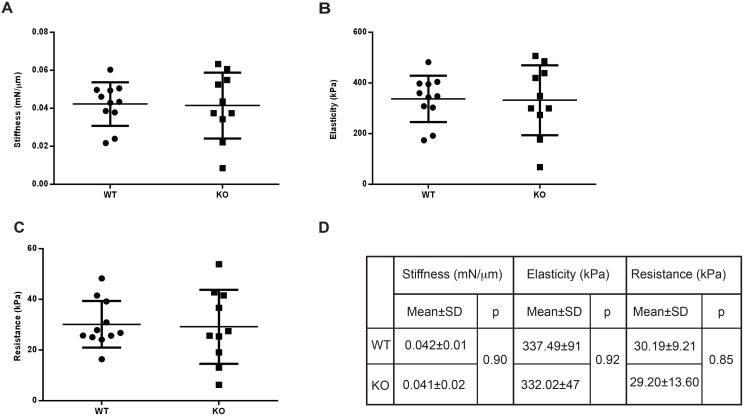
Biomechanical testing of cartilage. Articular surfaces were tested to measure (A) stiffness, (B) elasticity, and (C) resistance to penetration. Numerical values are shown (D) and statistical significance calculated with Student’s t test with p<0.05 seen to be significant, n = 10 WT and 10 KO.

An alternative explanation for the *Del1* KO mouse phenotype was simply that the cartilage was structurally weaker. Biomechanical testing was performed on the cartilage of the femoral head. The femoral head was chosen for analysis instead of the knee because the surface of the mouse knee joint was too small for adequate, reproducible measurements. We used 10 WT and KO male mice at 10 weeks of age for these studies. Specimens were analyzed using a microprobe system for stiffness, elasticity and resistance to penetration. No significant differences were seen in any of these parameters (Fig 5).

## Discussion

Despite the expression of *Del1* mRNA within cartilaginous structures during development and in the antenatal period, *Del1* KO mice were not different in the bony skeleton. We did note the KO mice had floppy ears noticeable primarily in the first weeks of life due to decreased thickness of the auricular cartilage. Additional analysis of the knee joints showed there was also diminished cartilage there. The finding that both elastic and hyaline cartilage, the two major types within the body, were decreased led us to conclude that there was a general decrease in the amount of cartilage due to this mutation.

Using primary chondrocytes, we examined how DEL1 might affect their biology to result in this phenotype. We found that DEL1 promoted chondrocyte attachment and was strongly anti-apoptotic. It had no impact on chondrocyte proliferation. Given the importance of apoptosis in the development of OA and the significant expression of *Del1 mRNA* within cartilage, we proposed the *Del1* KO mice develop more severe OA when compared to WT. We chose medial meniscectomy as a rapid and consistent trigger of post-traumatic OA as a model. Our data show *Del1* KO mice had more severe OA in response to injury and this was correlated with increased apoptosis within chondrocytes in those areas. Among the proteins that induced *Del1* mRNA expression, we found inflammatory mediators were the most prominent. These data led us to conclude that the phenotype was due to a positive survival signal provided by Del1 to chondrocytes, and may be a protective mechanism during periods of inflammation.

While we found increased chondrocyte apoptosis, there are a myriad other ways in which loss of DEL1 might lead to more severe OA. We examined a number of variables including angiogenesis, inflammatory cell infiltrate and biomechanical properties and found that we could not detect any significant differences. One limitation of these data is the unclear impact of the thinner cartilage found in *Del1* KO mice, but we did find no difference in the biomechanical properties suggesting the primary function of joint cartilage in permitting smooth locomotion was not affected. We clearly note our work may not be able to detect more subtle effects, but our studies do point to the fact that preventing apoptosis was a major contributor to the phenotype.

*Del1* KO mice are unique compared to most genetic mutants that have increased susceptibility to OA because they are grossly normal with the exception of a “floppy ear” phenotype early in life. Among the genetic mouse models of OA described,[7] mutations in major developmental regulatory genes typically required conditional knockouts due to embryonic lethality (i.e. *HH*).[13] Mutations in ECM proteins like COL2A1 display a variety of congenital malformations of the skeleton mirroring human pedigrees of patients with chondrodysplasias.[7] There are lines of mice that develop osteoarthritis spontaneously (SRT/Ort), but it is noted that this is not typical of human disease.[31] *Del1* KO mice develop more severe OA than WT after an inciting trauma. This is similar to the clinical experience in humans where individuals suffering the same injury have very different outcomes with regards to development of OA, and we suggest that the *Del1* KO mice represent a genetic model of susceptibility to OA that more closely mirrors the most common form of the human disease.

Previous genetic studies of non-syndromic OA susceptibility have indicated multiple genes contribute.[4–6] Our data suggest a recessive, single gene trait that is not readily recognized due to the subtle nature of the phenotype can cause more severe OA in response to trauma. Interestingly, a recent review of translational studies in OA specifically noted the post-traumatic model of OA used in our study most closely mimics actual human disease rather than those genetic mouse models that develop OA spontaneously, and this can have in impact in translational studies of new therapeutics.[32]

The role of apoptosis in the development of OA is well established. There are other mouse mutations described that lead to increased apoptosis of the articular chondrocytes. Mice with an inactivating point mutation in SIRT-1 had increased apoptosis of the chondrocytes, but were also noted to have reduced ECM components unlike the *Del1* KO mice.[15] CHOP is a down stream target of the unfolded protein response (UPR) activated during periods of cellular stress that functions to regulate multiple components of the apoptotic pathway. In *Chop-/-* mice, there was decreased severity of OA following knee destabilization associated with decreased apoptosis.[16] The *Chop-/-* mice provide an interesting parallel to the *Del1* KO mice in that both have a normal appearance with a phenotype expressed only after knee destabilization.

These data also show interesting parallels with the CCN family of matricellular proteins. Like DEL1, CCN proteins are ECM-associated and function in part through integrin binding. CCN1 promotes chondrocyte proliferation and aggregation, but has no known impact on skeletal development.[33,34] *Ccn2* deletion leads to embryonic lethality due to severe skeletal dysmorphisms by impairing chondrocyte proliferation and ECM production.[35] Mutations in *Ccn6* cause progressive pseudorhematoid dysplasia in humans, but have no impact on mouse skeletal development.[36,37] While DEL1 and CCN family members bind the same integrins, it is unclear what their relationship is to each other. Furthermore, the presence of multiple functional domains in these proteins increases the complexity of potential interactions.

Most OA therapies, including non-steroidal anti-inflammatory drugs (NSAIDs), behavior modification (weight loss, exercise) and surgical joint replacement, are to control OA symptoms rather than target the causal factors of the disease.[1,38] NSAIDs are capable of preventing inflammation, a significant component in the development of OA, but there are no data that NSAIDs can slow progression. A number of nutriceuticals (glucosamine/chondroitin) that looked promising in small studies have failed in larger, randomized trails to demonstrate efficacy at slowing OA disease progression.[39] Among the proteins identified which can contribute to OA, there are many that are less desirable targets for therapy because they are master regulatory proteins, and manipulation of their activity could have a host of unwanted side effects. Targeting enzymes involved in cartilage ECM degradation like MMPs is an active field of investigation. We have demonstrated that DEL1 protein was protective against OA, which suggests a new approach to OA treatment by using compounds that can directly block chondrocyte apoptosis.

## Supporting Information

S1 Fig Cell density of WT and KO cartilage. Calculated density of cells in cartilage of WT and KO mice performed by counting numbers of cells per high power field. N = 4 WT and KO mice.
(PDF)

S2 Fig Effect of DEL1 on chondrocyte proliferation. Normal human chondrocytes were cultured on plates coated with 8 ng/mm2 of BSA (DEL1-) or DEL1 (DEL1+) and proliferation assayed using WST-8 assay with absorbance read at OD450nm.
(PDF)

S3 Fig Immunohistochemistry for markers of angiogenesis and inflammation. Knee joints harvested at 8 weeks following medial meniscectomy were stained for CD31 as a marker of angiogenesis and at 1 week following medial meniscectomy for markers of lymphocytes (CD45R), macrophages (F4/80) and neutrophils (Ly-6B.2). Images shown are representative micrographs taken at 100x magnification. Positive cells per high power field were counted in the area of the medial compartment synovium and reported in the table below.
(PDF)
