# Supplementary figures and images for: Del1 Knockout Mice Developed More Severe Osteoarthritis Associated with Increased Susceptibility of Chondrocytes to Apoptosis
Source: PLoS One. 2016 Aug 9;11(8):e0160684. doi: 10.1371/journal.pone.0160684 (PMC4978450; doi:10.1371/journal.pone.0160684)

Supplemental Figure 1

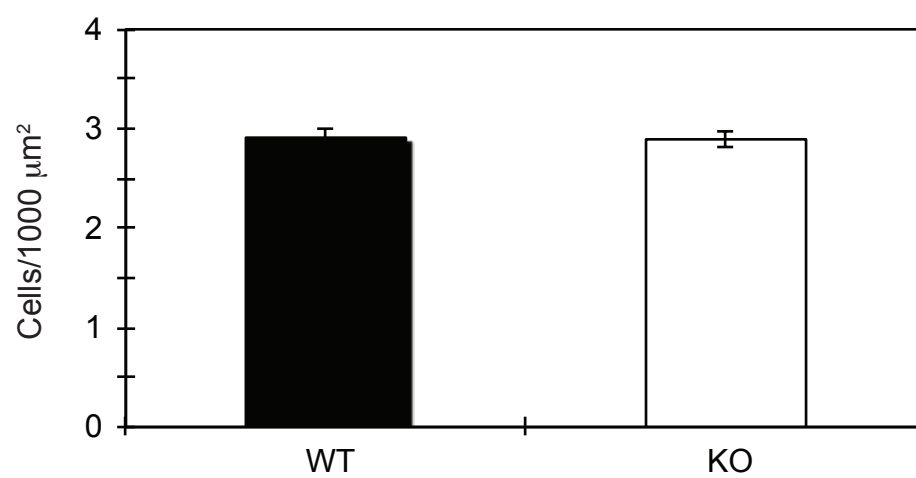

Supplement: S1 Fig — Calculated density of cells in cartilage of WT and KO mice performed by counting numbers of cells per high power field. N = 4 WT and KO mice. (PDF) [file pone.0160684.s001.pdf]

Supplemental Figure 2

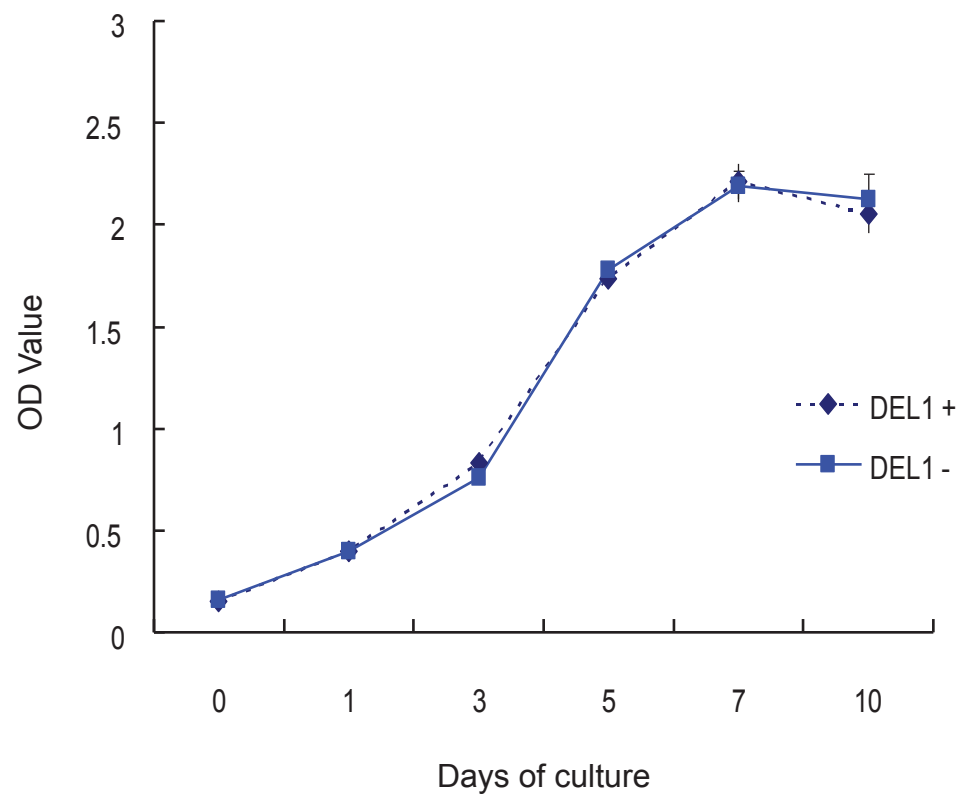

Supplement: S2 Fig — Normal human chondrocytes were cultured on plates coated with 8 ng/mm2 of BSA (DEL1-) or DEL1 (DEL1+) and proliferation assayed using WST-8 assay with absorbance read at OD450nm. (PDF) [file pone.0160684.s002.pdf]

Supplemental Figure 3

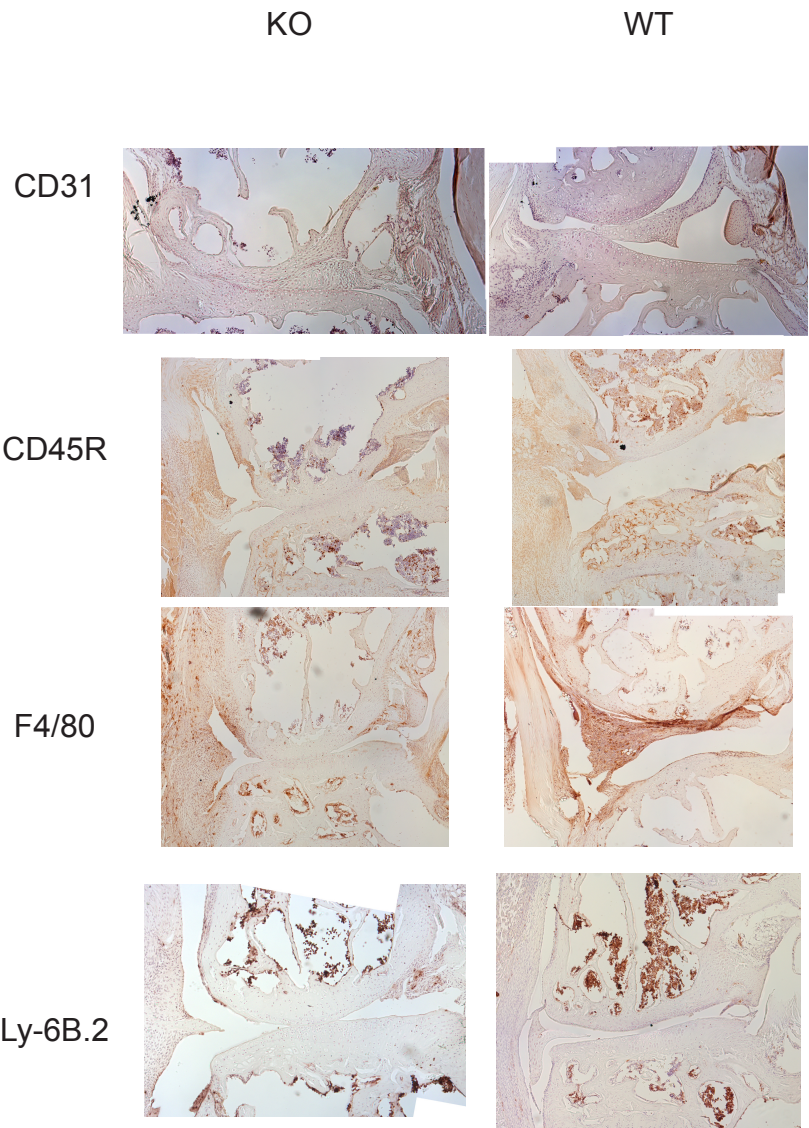

|         | WT    |      | KO    |      |      |
|---------|-------|------|-------|------|------|
|         | cells | SD   | cells | SD   | p    |
| CD31    | 0     | -    | 0     | -    | -    |
| CD45R   | 0     | -    | 0     | -    | -    |
| F4/80   | 12.33 | 1.24 | 9.00  | 2.16 | 0.16 |
| Ly-6B.2 | 10.67 | 6.80 | 7.50  | 2.06 | 0.59 |

Supplement: S3 Fig — Knee joints harvested at 8 weeks following medial meniscectomy were stained for CD31 as a marker of angiogenesis and at 1 week following medial meniscectomy for markers of lymphocytes (CD45R), macrophages (F4/80) and neutrophils (Ly-6B.2). Images shown are representative micrographs taken at 100x magnification. Positive cells per high power field were counted in the area of the medial compartment synovium and reported in the table below. (PDF) [file pone.0160684.s003.pdf]
